# Evaluating circulating cell-free DNA and DNA integrity index as biomarkers in non-small cell lung cancer

**DOI:** 10.1186/s43046-024-00219-1

**Published:** 2024-06-17

**Authors:** Nada Ezzeldin, Dalia El-Lebedy, Mirhane Hassan, Alaa Omar Shalaby, Sabah Ahmed Mohamed Hussein, Ahmed Mohamed Gharib, Gehan Hamdy, Asmaa Mahmoud Mohammed, Abeer Ramadan, Mohamed Emam Sobeih

**Affiliations:** 1 Chest Diseases, National Research Centre, Cairo, Egypt; 2 Clinical Pathology department, Medical Research and Clinical Studies Institute, National Research Centre, Cairo, Egypt; 3 Chest Diseases, Faculty of Medicine, Cairo University, Cairo, Egypt; 4 Department of Environmental and Occupational Medicine, National Research Centre, Cairo, Egypt; 5 Molecular Genetics and Enzymology Department, Human Genetics and Genomics Research Institute, National Research Centre, Cairo, Egypt; 6 Oncology, National Cancer Institute, Cairo University, Cairo, Egypt

**Keywords:** Circulating tumour DNA, Circulating cell-free DNA, NSCLC, DNA integrity index, ALU247, ALU115

## Abstract

**Background:**

Analysis of free DNA molecules shed from tumour cells in plasma of patients referred as circulating tumour DNA (ctDNA) with reference to physiological circulating cell-free DNA (cfDNA) is nowadays exploited as liquid biopsy and is considered a new emerging promising biomarker for diagnosis, selection of proper treatment, and prognosis of cancer. DNA integrity index (DII) is assessed by calculating the ratio between the concentration of long cfDNA strands released from tumour cells (ALU247) and the short strands released from normal cells (ALU115). The aim of the current study was to evaluate DII as a potential diagnostic and prognostic biomarker of NSCLC.

**Methods:**

Our study included 48 NSCLC patients diagnosed as primary NSCLC before starting treatment, 30 COPD patients diagnosed clinically, radiologically, and subjected to chest high-resolution computerized tomography, and 40 healthy controls. cfDNA concentration and DII were measured by quantitative real-time polymerase chain reaction (qPCR).

**Results:**

ALU115, ALU247, and DII were significantly higher in NSCLC compared to COPD patients (*p* < 0.0001) and controls (*p* < 0.0001) and in COPD patients compared to control subjects (*p* < 0.0001). DII positively correlated with the stage of tumour (*p* = 0.01), tumour metastasis (*p* = 0.004), and with adenocarcinoma compared to other histopathological types (*p* = 0.02). To evaluate clinical utility of DII in NSCLC, ROC curve analysis demonstrated an AUC of 0.91 at a cut-off value of 0.44 with total accuracy = 85.6%, sensitivity = 90%, specificity = 83%, *PPV* = 78.1%, and *NPV* = 92.1%.

**Conclusion:**

cfDNA and DII represent a promising diagnostic and prognostic tool in NSCLC. This type of noninvasive liquid biopsy revealed its chance in the screening, early diagnosis, and monitoring of NSCLC.

## Background

Lung cancer, especially non-small cell lung cancer (NSCLC) that accounts for 80–85% of lung cancer cases, represents the foremost lethal malignancy in Egypt; however, it is the fourth most prevalent cancer in men, and it is relatively rare in women [1]*.* The latest WHO data published in 2020 that lung cancer mortality represented 1.06% of total deaths in Egypt, with an age-adjusted death rate of 8.02 per 100,000 of population, ranking Egypt no. 115 within the world [2]*.*

Cancer screening and early detection tests are of critical importance to enhance treatment effectiveness and subsequently decrease cancer morbidity and mortality rate [3]. Currently, tissue biopsies are considered the gold standard for tumour profiling. However, this method in addition of being invasive, risky, and not easily obtained for a few anatomical locations, it provides an incomplete analysis of the tumour profile due to tumour heterogeneity that requires multiple biopsies. Moreover, biopsies can hardly detect discrepancies between primary and metastatic lesions, beside its inability to detect the continuous genetic changes that may result in new mutations and subsequent resistance to treatment, hence the ineffectiveness of biopsies to observe disease progression [4].

Researches on liquid biopsies using different biological fluids of patients provide genomic [5], epigenetic [6], proteomic, and transcriptomic [7] evidence about tumours and metastasis. The clinical utilization of liquid biopsies has been suggested to improve cancer screening [8], diagnosis [9], prognosis [10, 11], monitoring [12], assessing treatment response [13, 14], and detecting treatment-resistant clones [15].

Circulating cell-free DNA (cfDNA) is believed to be the most hopeful biomarker, being the simplest to perform in clinical routine, and various applications have been developed for diagnostic, therapeutic, and prognostic purposes [16]*.* A portion of this DNA is shed from tumour cells into the blood stream and referred to as circulating tumour DNA (ctDNA). In healthy individuals, apoptosis of cells generates short fragments of DNA < 200 bp [17, 18], while necrosis of tumour cells results in shedding of longer DNA fragments [17]. The *Arthrobacter luteus* (ALU) repeats, 300 bp long, are the most prevailing repeated sequences all over the human genome. They account for more than 10% of the human genome [19]. The ratio between long- and short-generated DNA fragments, ALU247 and ALU115, respectively, is referred to as DNA integrity index (DII) [20].

A significantly increased DII was demonstrated in colorectal cancer [18], hepatitis C virus-related hepatocellular carcinoma [21], breast cancer with a positive correlation with TNM staging [22, 23], and in bronchogenic carcinoma [24–27].

The aim of this study was to evaluate the clinical utility of DII as a potential noninvasive biomarker in NSCLC and its role in diagnosis and prognosis.

## Methods

### Subjects

This work was done in National Research Centre (NRC) in cooperation with National Cancer Institute (NCI) and Chest Department of Cairo University Hospital (Kasr-ElAiny). This case–control study included 48 unrelated adult patients with primary lung cancer, 30 COPD patients, and 40 unrelated controls. All subjects included in the study were questioned about the lifetime smoking history, residence, occupational history, and family history of cancer. Detailed clinical examination and chest radiographic evaluation, i.e. high-resolution computerized tomography (HRCT), were carried out for diagnosis of COPD patients. Specimens obtained either by open biopsy or via bronchoscopy were assessed for lung cancer by histopathological evaluation. Venous blood sample was drawn from each subject to measure the absolute concentration of ALU247 and ALU115 and to calculate DII. Patients’ inclusion criteria were primary lung cancer confirmed by histopathology, age ≥ 18, and anti-tumour therapy either surgery, chemotherapy, or radiotherapy has not been started yet. Previous history of cancer, metastasis from other organs, pulmonary fibrosis, acute interstitial pneumonia, radio/chemotherapy, or any anticancer therapy received anytime earlier than the study were all considered exclusion criteria for the patients.

The study was permitted by the Ethics Committee of the National Research Centre, registration number 19–234. Informed written consent was obtained from all subjects after being fully aware of the nature of the study.

### Samples collection and processing

A total of 5-ml venous blood samples were collected in EDTA vacutainers from newly diagnosed lung cancer patients, COPD patients, and controls and centrifuged at 3000 rpm for 10 min at 4 °C, then plasma layer was removed carefully and spun again for further 5 min at 2500 rpm for better purity, and, finally, plasma was put in storage at − 80 °C until PCR analysis.

### Measurement of cfDNA concentration by quantitative real-time PCR

cfDNA was extracted from plasma using the QIAamp DNA Mini kit (Qiagen, Hilden, Germany) according to manufacturer’s instructions. cfDNA concentrations in plasma samples were evaluated by measuring ALU115 and ALU247 repeats. All primers were supplied from Invitrogen (Thermo Fisher, Scientific Inc., USA).

Primers’ sequences were ALU115 forward: 5′-CCTGAGGTCAGGAGTTCGAG-3′ and reverse: 5′-CCCGAGTAGCTGGGATTACA-3′ and ALU247 forward: 5′-GTGGCTCACGCCTGTAATC-3′ and reverse: 5′-CAGGCTGGAGTGCAGTGG-3′.

Quantitative real-time PCR was conducted on StepOne real-time PCR (Applied Biosystems, Foster City, CA, USA) using FastStart Universal SYBR Green Master Mix (Life Technologies-Thermo Fisher, Scientific Inc., USA). PCR amplification was performed with pre-activation of DNA polymerase at 95 °C for 15 min, followed by 40 cycles of denaturation at 94 °C for 15 s, annealing at 64 °C for 30 s, extension at 72 °C for 30 s, and extension at 72 °C for 10 min.

Standard curves for ALU-247 and ALU-115 were obtained using human genomic DNA control PCR-ready concentration (Applied Biosystems) with serial dilutions from 10 ng/μL to 0.01 pg/μL to calculate the absolute concentration of ALU247 and ALU115 in each sample. Integrity index was calculated as ALU247/ALU115.

### Statistical analysis

Collected data and clinical results were tabulated and statistically analysed using IBM SPSS version 20.0 software (Statistical Package for Social Science). Quantitative data were expressed as mean ± standard deviation (SD) for normally distributed data and compared using one-way ANOVA test for multiple comparisons and Student’s *t*-test for two groups comparison. Post hoc test has been conducted following ANOVA test to determine the differences between pairs within the groups. The qualitative data were expressed as number and percentage and then compared using the chi-square test (*χ*^2^). Skewed data were expressed as median and interquartile range (IQR) and compared using Mann–Whitney *U*-test for two groups and Kruskal–Wallis test for multiple groups. Donn’s test has been conducted following Kruskal–Wallis test to determine the differences between pairs within the groups. Point-biserial correlation (r^pb^) analysis has been used to find potential correlation between the integrity index and tumour characteristics. ROC curve analysis was used to assess the validity of variables as diagnostic tools (95% CI). *p*-value < 0.05 was considered significant (2-tailed).

## Results

**Fig. 1:**
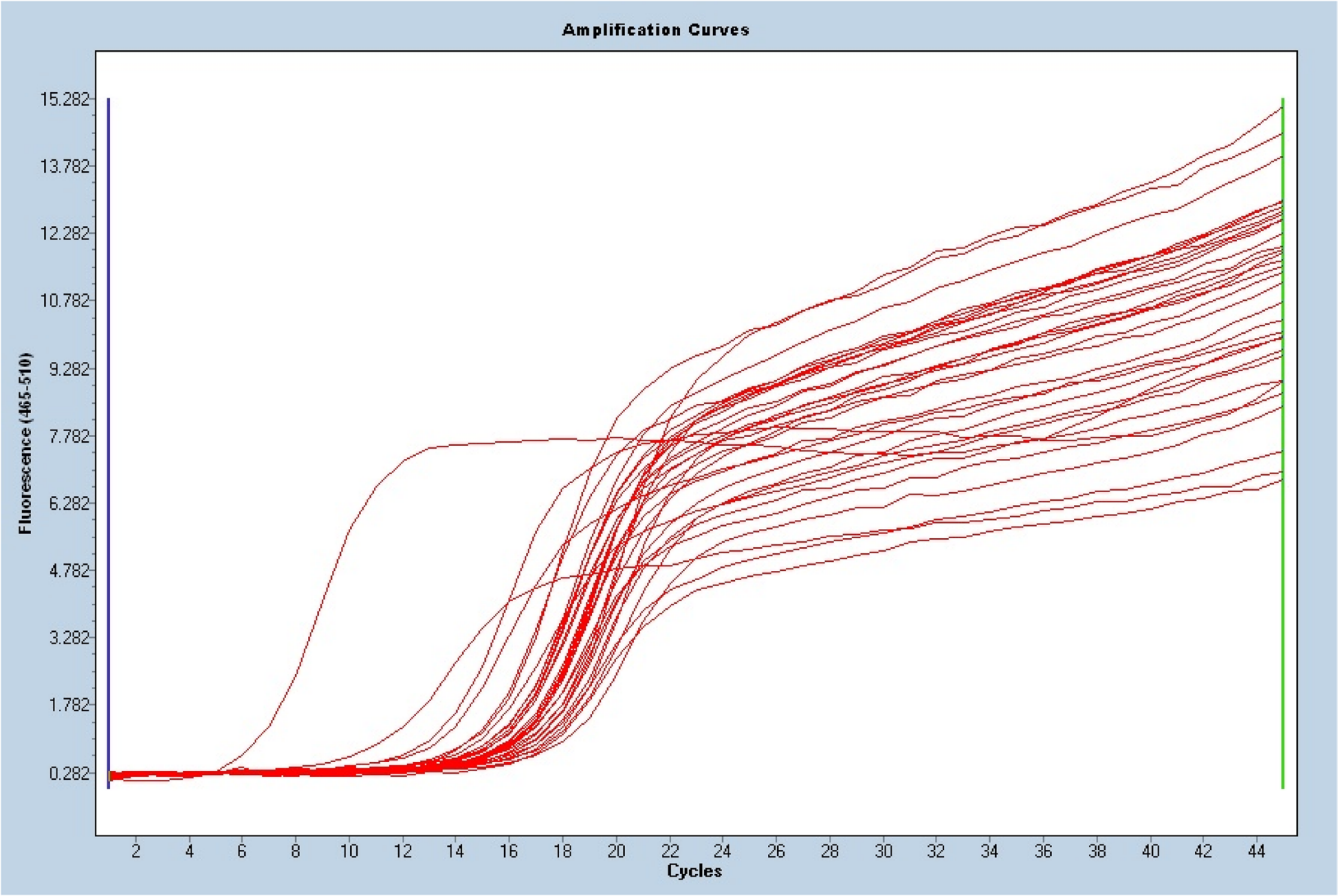
qPCR amplification curve of ALU 115

**Fig. 2:**
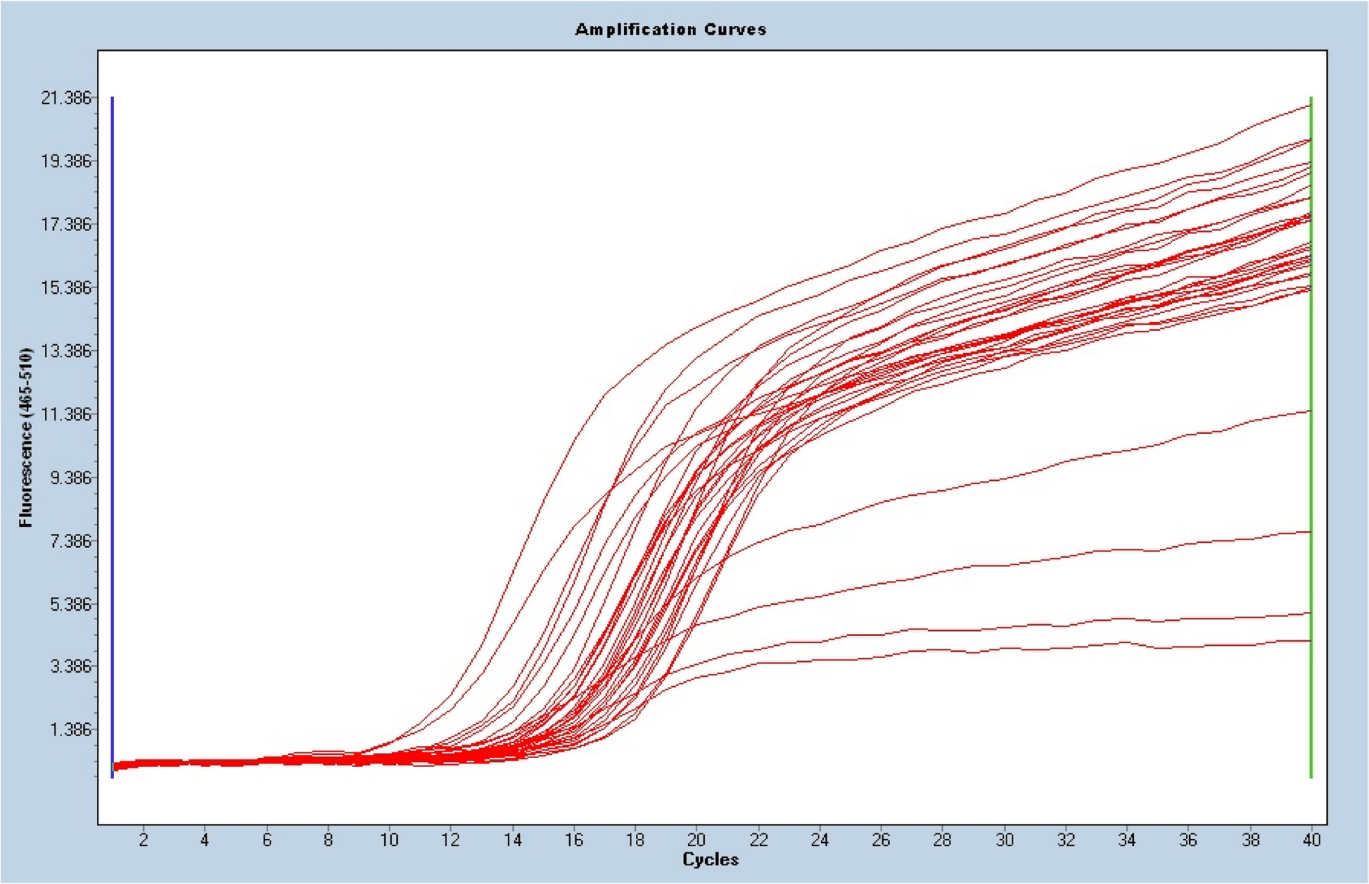
qPCR amplification curve of ALU 247

**Fig. 3:**
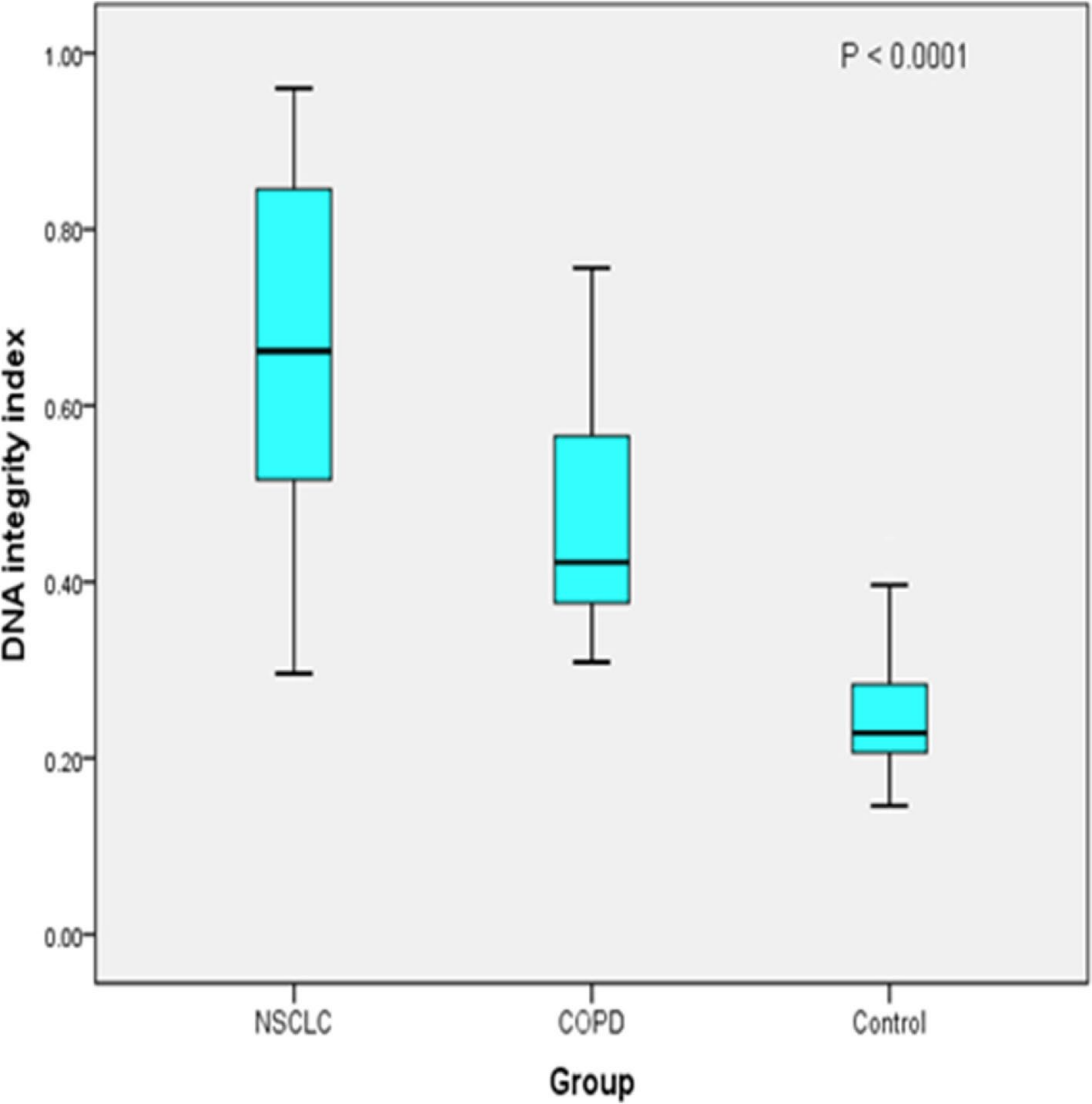
DNA integrity index (DII) in the three studied groups

A total number of 118 individuals have been studied with mean age 54.8 ± 10.9 and age range (24–85 years). They were classified into 48 patients with lung cancer with mean age 58.6 ± 11.1, 30 patients with COPD with mean age 57.7 ± 9.0, and 40 healthy control with mean age 48.0 ± 9.0 years. All groups were matched in sex (*p* > 0.05). Significant higher frequencies of smoking and pack year index were observed among cancer and COPD patients than in control group (*p* < 0.0001). Significant higher values of ALU247 and DII were detected in lung cancer patients compared to COPD patients and control group (*p* < 0.0001) and in COPD patients than in control group (*p* < 0.0001) (Figs. 1 and 2) (Table 1). Comparison of integrity index among the three populations (lung cancer, COPD, and controls) is shown in Fig. 3.

**Table 1:** General characteristics of different studied groups

	**Lung cancer (*n* = 48)**	**COPD (*n* = 30)**	**Controls (*n* = 40)**	***p*-value**
**Age (years)**				
Mean ± SD	58.6 ± 11.1	57.7±9.0	48.0 ± 9.0	< 0.0001*
Range	(24–85)	(37–70)	(28–68)	
**Gender *n* (%)**				
Male	42 (87.5)	30 (100.0)	36 (90.0)	0.1
Female	6 (12.5)	0 (0.0)	4 (10.0)	
**Smoking status *n* (%)**				
Smoker	38 (79.2)	30 (100.0)	20 (50.0)	< 0.0001*
Non-smoker	10 (20.8)	0 (0.0)	20 (50.0)	
**Pack-years index**				
Median)IQR)	45 (30–63.7)	55 (33.7–80)	21.2 (17.1–30)	< 0.0001*
**ALU-115 ng/ml**				
Median)IQR)	77.4 (48.8–114)	36 (16.7–62.1)	6.8 (5.8–7.6)	< 0.0001*^≠ ˦^
**ALU-247 ng/ml**				
Median)IQR)	52.1 (28.6–89.9)	16.8 (6.6–31.6)	1.6 (1.4–1.9)	< 0.0001*^≠ ˦^
**DII**				
Median (IQR)	0.6 (0.5–0.8)	0.4 (0.3–0.5)	0.2 (0.22–0.3)	< 0.0001*^≠ ˦^

*IQR* interquartile range

*Lung cancer vs. controls

^≠^Lung cancer vs. COPD

^˦^COPD vs. controls

ALU247 was significantly higher in patients with metastasis (stage IV) than in early stages (stages I and II) (*p* = 0.04) or in advanced stage (stage III) (*p* = 0.01). In respect to histopathological type, adenocarcinoma patients showed significantly higher values of ALU247 and DNA integrity than other histopathological types (*p* = 0.04 and 0.02, respectively). No significant difference was found between different grades of lung cancer. Correlation analysis showed significant positive correlations between DNA integrity index and stage of tumour (*p* = 0.01) and tumour metastasis (*p* = 0.004) (Tables 2 and 3). DII in healthy smokers of the control group showed significant higher mean 0.28 ± 0.07 in comparison to the healthy nonsmokers in the same group 0.22 ± 0.06 (*p* = 0.01).

**Table 2:** ALU115 and ALU247 in relation to tumour characteristic among lung cancer group

	***N* (%)**	**ALU115 (mean ± SD)**	***p*-value**	**ALU247 (mean ± SD)**	***p*-value**	**DII (mean ± SD)**	***p*-value**
**Clinical stage**							
Stage (I & II)	5(10.4)	55.2 ± 12.9	0.014*	33.4 ± 6.5	0.012*	0.6 ± 0.03	0.012*
Stage III	11(22.9)	58.9 ± 36.7		36.6 ± 36.4		0.5 ± 0.17	
Stage IV	32(66.7)	104.8 ± 56.9		84.4 ± 58		0.7 ± 0.18	
**Histopathology**							
Adenocarcinoma	29(60.4)	102.4 ± 53.2	0.03*	81.2 ± 56.1	0.04*	0.7 ± 0.1	0.02*
Other (SCC, LCC)	19(39.6)	68.9 ± 50.5		48.2 ± 48.7		0.6 ± 0.2	
**Grade**							
I	3(6.2)	61.9 ± 12	0.8	36.5 ± 7	0.9	0.6 ± 0.01	0.6
II	2(4.2)	45.1 ± 6.3		28.8 ± 2.6		0.6 ± 0.03	
III	11(22.9)	58.9 ± 36.7		36.6 ± 36.4		0.5 ± 0.17	
**Metastatic**	32(66.7)	104.8 ± 56.9	0.003*	84.4 ± 58	0.003*	0.7 ± 0.18	0.004*
**Non-metastatic**	16(33.3)	57.7 ± 30.7		35.6 ± 29.9		0.5 ± 0.1	

*SCC* squamous cell cancer, *LCC* large cell carcinoma

^∗^Significant at the level⩽ 0.05 (2-tailed)

**Table 3:** Correlation analysis between DNA integrity index and tumour characteristics

Tumour characteristic	*r*^pb^	*P*
Tumour metastasis	0.4	0.004*
Tumour stage	0.3	0.01*
Tumour grade	0.1	0.5
Adenocarcinoma vs. other types	0.3	0.01*

^∗^Significant at the level⩽ 0.01 (2-tailed)

**Fig. 4:**
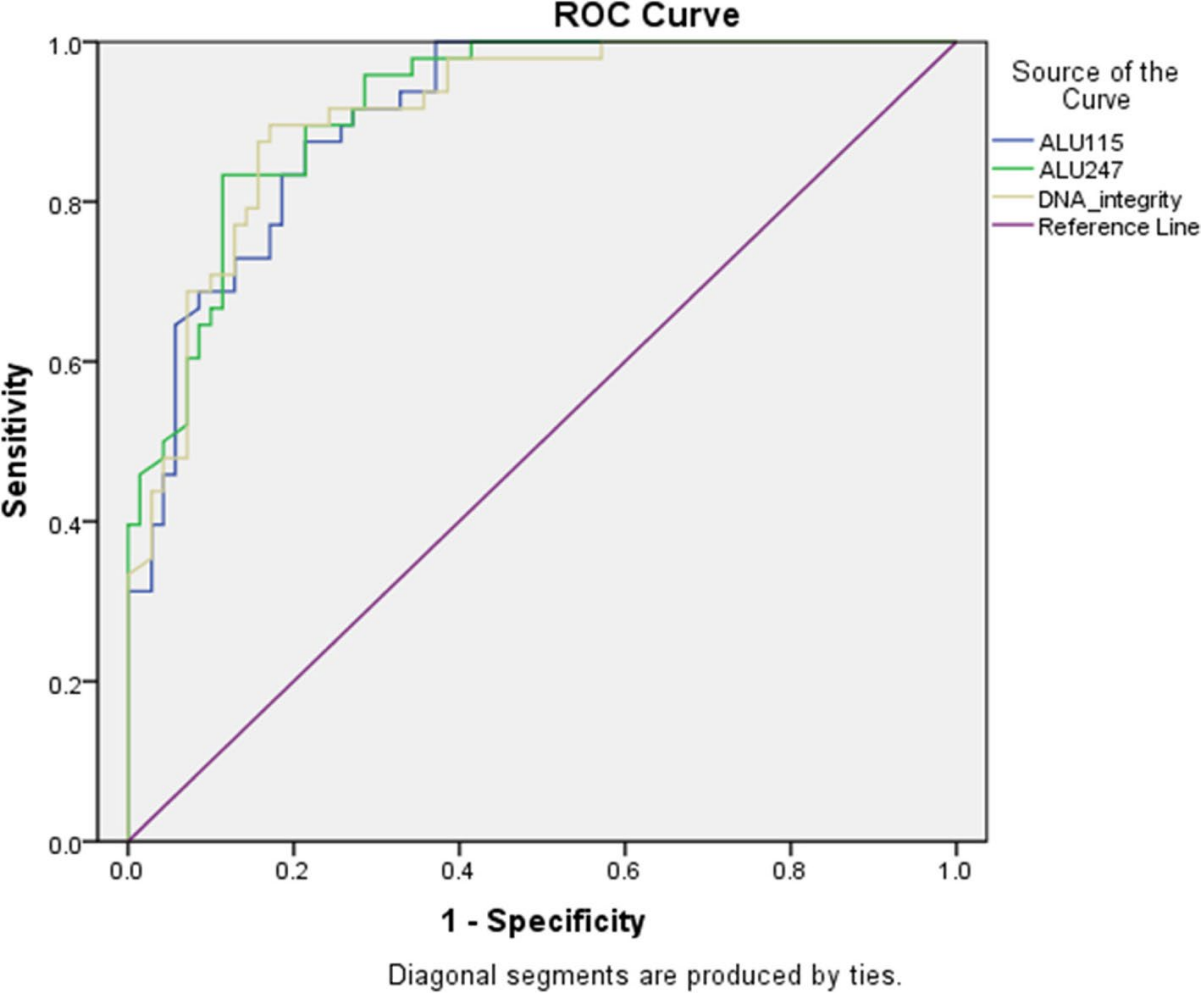
ROC curve analysis of ALU115, ALU247, and DNA integrity index as diagnostic markers for lung cancer

ROC curve analysis showed an AUC for *ALU247* = 0.92 with total accuracy = 86.4% at a cut-off value of 26.1 ng/ml with sensitivity = 83%, specificity = 89%, positive predictive value (PPV) = 83.3%, and a negative predictive value (NPV) = 88.5 (*p* < 0.0001). For DNA integrity, the AUC was 0.91 with total accuracy of 85.6% at a cut-off value 0.44 ng/ml, with sensitivity = 90%, specificity = 83%, *PPV* = 78.1%, and *NPV* = 92.1 (*p* < 0.0001) (Fig. 4).

## Discussion

This study aimed to evaluate the clinical utility of DII as a biomarker in NSCLC in comparison with COPD and controls.

Previous studies reported a significantly increased DII in different types of cancer indicating its value as a diagnostic marker*.* In the current study, we assessed the utility of DII as a diagnostic tool in lung cancer patients in comparison with COPD and control subjects and studied its potential correlation with tumour stage and metastasis. Our results demonstrated significantly higher integrity index in NSCLC patients compared to COPD patients and control subjects. These results are in agreement with previous results [28–33]. Moreover, significant higher values were observed in COPD patients compared to the control group. In accordance with the findings of [30] who reported significant differences in ALU115, ALU247 concentrations, and DII between COPD and control groups, which was attributed to inflammation contributing to increased cell death rates [34]. In contrast, *Soliman *et al*. (2018)* found insignificant differences in ALU115, ALU247, and DII between COPD and control groups [33].

According to TNM staging system, a significantly high ALU115, ALU247, and DII were detected among late-stage cancer patients (III & IV) and in cases with metastatic lesions in comparison with non-metastatic cases. Adenocarcinoma showed significantly higher values of all parameters compared to other histopathological types (squamous and large cell carcinoma). Findings are in line with similar studies which reported higher plasma concentration of cfDNA in late stages of tumour than in early stages [35, 36] and more elevation in metastatic than in non-metastatic cases [32, 33]. In contrast, other researches could not find any association between cfDNA concentration and tumour stage [37], metastasis [38], or histological type (adenocarcinoma or squamous cell carcinoma) [31].

*Wang *et al*. (2003)* declared DII clinical utility by proving significantly higher value in ovarian and gynaecological cancers [39]. Furthermore, studies in solid tumuors such as colorectal [18, 40, 41], breast [17, 42], head and neck [43], and prostate cancers [44] reported positive results for diagnostic and prognostic value of ALU repeats. That might be elucidated by vascular invasion occurring in malignant tumours that enhances DNA release into the circulation, hence enabling the dissemination of tumour cells [35].

*Francaviglia *et al*. (2019)* investigated lung cancer cfDNA analysis and reported the successful use of plasma ctDNA for identification of therapeutic targets, monitoring of therapy, and resistance to treatment [27], while *Dziadziuszko *et al*. (2022)* suggested the usefulness of plasma cfDNA concentration as a prognostic marker in advanced *ALK* + NSCLC [45].

To evaluate diagnostic validity of integrity index, ROC curve analysis demonstrated a cut-off value of 0.44 with total accuracy of 85.6%, sensitivity and specificity 90% and 83%, respectively, and PPV 78.1% and NPV 92.1. The median of DNA integrity in COPD cases in this study was 0.4 ng/ml (*IQR* = 0.3–0.5 ng/ml).

In a similar study on 60 NSCLC patients 40 COPD and 40 controls, *Soliman *et al*. (2018)* demonstrated a cut-off value of 0.48 ng/ml for DNA integrity but with lower sensitivity and specificity, 75% and 42.5%, respectively [33]. They also found that ALU247 had higher diagnostic accuracy 92.1%, with sensitivity and specificity of 96.7% and 88.7%, respectively, and PPV of 86.6% and NPV of 97.3%. Similarly, in a study on 50 NSCLC patients, 101 patients with chronic respiratory inflammation, and 40 healthy controls, *Szpechcinski *et al*. (2015)* found that the use of the quantitative cfDNA assay as a diagnostic biomarker to differentiate NSCLC from non-malignant inflammatory diseases and healthy individuals showed a highest accuracy achieved at a cut-off value of 42.80 ng/ml, with sensitivity of 90% and specificity of 80.5% and PPV of 85% and NPV of 87% [31].

*Kamel *et al*. (2016)* reported a significantly higher level of DII in plasma of breast cancer than patients with in benign breast lesions and healthy controls. They stated a sensitivity and specificity values of DII 85% and 100%, respectively, defining the clinical utility of ctDNA. Moreover, they described a correlation between DII and TNM staging suggesting that DII might be a promising diagnostic and prognostic biomarker of breast cancer [23].

In a previous study to check the accuracy, sensitivity, and specificity of using cfDNA as a screening tool in cancer patients against both benign and control groups, cfDNA represented a highly sensitive and specific marker, 100% and 75%, respectively, in comparison to traditional tumour markers [38].

In a recent study on 84 NSCLC patients, *Ren *et al*. (2023)* reported a significantly higher concentration of plasma cfDNA (ALU60, ALU115) in NSCLC patients (stage III/IV) than in healthy controls [46].

In contrast, other studies concluded that DII is not a useful clinical tool to detect malignancies because of its poor specificity and sensitivity [47, 48]. Discrepancies noticed among different studies might be related to the variation in selection of subjects, pre-analytical and analytical procedures, and to the characteristics and numbers of patient populations.

Despite the valuable implications of our results, this study has several limitations. First, the small sample size in addition to discrepancy of demographic characteristics (age/gender) and smoking behaviour between patients and control may introduce bias. Therefore, larger *cohort studies* with balanced demographics between NSCLC patients and controls are required in the future researches. Second, the study’s cross-sectional design limits its capacity to assess the prognostic value of cfDNA and DII over time. Third, our study lacks direct comparison with other established NSCLC biomarkers, hindering proper insights of cfDNA and DII’s significance. Fourth, we did not explore the potential influence of other factors such as comorbidities, medications, and environmental factors on cfDNA and DII levels. Finally, challenges in translating these findings into clinical practice necessitate further validation in diverse populations and clinical settings.

## Conclusion

As liquid biopsies represent a potential, attractive, noninvasive alternative of routine surgical biopsies for diagnosis and prognosis of cancer, this study aimed to evaluate the clinical utility of DII as a biomarker in NSCLC in comparison with COPD and controls.

By detecting cfDNA concentrations in plasma samples and evaluating DII by means of ALU repeats, we suggest that DII could be a candidate biomarker in the early detection and screening for NSCLC. We also identified a prognostic role of this new emerging biomarker, being correlated with TNM and metastasis, highlighting its potential role in monitoring disease progression. We also identified a cut-off value for DNA integrity index in COPD patients for periodic follow-up and closer monitoring to help screening and early detection of lung cancer among COPD patients.

## Data Availability

All data supporting our findings can be found in the "Results" section in the main manuscript.

## Abbreviations

ALU: *Arthrobacter luteus*
cfDNA: Circulating cell-free DNA
COPD: Chronic obstructive lung disease
ctDNA: Circulating tumour DNA
DII: DNA integrity index
EDTA: Ethylenediaminetetraacetic acid
HRCT: High-resolution computerized tomography
IQR: Interquartile range
LCC: Large cell carcinoma
NCI: National Cancer Institute
NPV: Negative predictive value
NRC: National Research Centre
NSCLC: Non-small cell lung cancer
PPV: Positive predictive value
qPCR: Quantitative real-time polymerase chain reaction
SCC: Squamous cell carcinoma
SD: Standard deviation
TNM: Tumour, nodes, and metastasis
WHO: World Health Organization
